# Relationship between temporal dynamics of intrinsic brain activity and motor function remodeling in patients with acute BGIS

**DOI:** 10.3389/fnins.2023.1154018

**Published:** 2023-06-27

**Authors:** Xiaoling Chen, Wenmei Li

**Affiliations:** Department of Radiology, The First Affiliated Hospital of Guangxi Medical University, Nanning, China

**Keywords:** acute basal ganglia ischemic stroke, resting-state fMRI, dynamic low-frequency fluctuation amplitude, dynamic intrinsic brain activity, motor function remodeling

## Abstract

**Background:**

patients with acute basal ganglia ischemic stroke (BGIS) show changes in local brain activity represented by the amplitude of low-frequency fluctuation (ALFF), but the time-varying characteristics of this local nerve activity are still unclear. This study aimed to investigate the abnormal time-varying local brain activity of patients with acute BGIS by using the ALFF method combined with the sliding-window approach.

**Methods:**

In this study, 34 patients with acute BGIS with motor dysfunction and 44 healthy controls (HCs) were recruited. The dynamic amplitude of low-frequency fluctuation (dALFF) was employed to detect the alterations in brain activity induced by acute BGIS patients. A two-sample *t*-test comparison was performed to compare the dALFF value between the two groups and a Spearman correlation analysis was conducted to assess the relationship between the local brain activity abnormalities and clinical characteristics.

**Results:**

Compared with HCs, the activity of neurons in the left temporal pole (TP), parahippocampal gyrus (paraHIP), middle occipital gyrus (MOG), dorsolateral superior frontal gyrus (SFGdl), medial cingulate cortex (MCC), right rectus, precuneus (PCu) and right cerebellum crus1 were significantly increased in patients with BGIS. In addition, we found that there was a negative correlation (*r* = −0.458, *p* = 0.007) between the dALFF value of the right rectus and the scores of the National Institutes of Health Stroke Scale (NIHSS), and a positive correlation (*r* = 0.488, 0.499, *p* < 0.05) with the scores of the Barthel Index scale (BI) and the Fugl Meyer motor function assessment (FMA). ROC analysis results demonstrated that the area under the curves (AUC) of the right rectus was 0.880, *p*<0.001.

**Conclusion:**

The pattern of intrinsic brain activity variability was altered in patients with acute BGIS compared with HCs. The abnormal dALFF variability might be a potential tool to assess motor function in patients with acute BGIS and potentially inform the diagnosis of this disease.

## Introduction

More than 80% of ischemic stroke is caused by acute reduction of blood supply caused by thrombus or embolus obstruction, which leads to cerebral ischemic injury (Krishnamurthi et al., 2018). When the blood flow drops below a critical level, the electrical function of neurons stops, and a series of neurological defects such as motor and sensory abnormalities occur (Hertelendy et al., 2017; Kessner et al., 2019). Motor dysfunction is one of the major disabilities of ischemic stroke, which seriously affects the quality of life of patients (Feigin et al., 2014). The clinical manifestations of ischemic stroke are related to specific sites of the central nervous system (Jin and Zhao, 2018). Because of active metabolism in basal ganglia, ischemic stroke often involves this area (Tambasco et al., 2018). Acute ischemic stroke occurring in the basal ganglia region is defined as acute basal ganglia ischemic stroke (BGIS). The basal ganglia region is mainly involved in motor execution and motor control processes (Roseberry et al., 2016; Florio et al., 2018). Dysfunction of the basal ganglia region is usually secondary to motor dysfunction (Cantello and Civardi, 2002). The recovery of behavioral function after ischemic stroke is largely due to neurological remodeling (Sinke et al., 2018). Therefore, it is important to explore the biological basis of motor function defects and the mechanism of motor function remodeling in patients with acute BGIS.

Resting-state functional magnetic resonance imaging (rs-fMRI) reflects the spontaneous activity of the human brain under physiological conditions, showing the damage and reorganization of brain spontaneous activity caused by ischemic stroke and the functional reorganization between brain regions (van Meer et al., 2010; Jiang et al., 2019; Gao et al., 2021). Recent resting-state fMRI studies on BGIS patients have mainly focused on the regional homogeneity (ReHo) and functional connectivity (FC) methods, they focus on the similarities of intra-and inter-regional time series, respectively, (Li X. et al., 2020; Li Q. G. et al., 2020; Xu et al., 2020; Li J. et al., 2022; Li Q. et al., 2022), while ignoring spontaneous local activity, a crucial characteristic for understanding the intrinsic functional architecture of brain (Fox and Raichle, 2007). The amplitude of low-frequency fluctuation (ALFF) is proposed to detect the blood oxygen level-dependent (BOLD) signal fluctuations, which can reflect the characteristics of spontaneous activity of local neurons in the resting state by calculating the power spectrum of low-frequency fluctuation signals with a frequency of 0.08–0.10 Hz (Yu-Feng et al., 2007; Tsai et al., 2014). Previous studies demonstrated ALFF has great potential in estimating the survival ability of brain tissue after ischemia (Yao et al., 2012; Tsai et al., 2014). Quan et al. (2022) applied ALFF for detecting alters in local brain of low-frequency oscillations (LFOs) in acute BGIS patients, such as the superior marginal gyrus (SMG), suggesting that local properties are crucial to comprehending the neural pathology of motor function reorganization associated with stroke.

Most of the above-mentioned rs-fMRI studies adopted the assumption that the brain activity remains‘static’, while the brain dynamic map reflects the temporal variability related to neural function (Yang et al., 2019). Therefore, ALFF only the average brain activity of the entire BOLD signal time series may lose a lot of subtle information about brain fluctuations (Lydon-Staley et al., 2019). Combining the ALFF with “sliding-window” approaches, the dynamic ALFF (dALFF) method was proposed to characterize the dynamic local properties of brain activity (Liao et al., 2019). Dynamic ALFF have effectively revealed the time-domain dynamic characteristics of spontaneous neuronal activity in patients with post-stroke aphasia (Guo et al., 2019) and post-stroke depression (Yao et al., 2020). In addition, study on ischemic stroke patients with dALFF also found significant changes in temporal variability of neural activity in multiple cortical regions, such as the cerebellum, Inferior temporal gyrus (IFG), dorsolateral superior frontal gyrus (SFGdl), and default mode networks (DMN), etc. (Chen et al., 2018; Tian et al., 2022; Wang et al., 2023). It has a potential compensation effect on the motor dysfunction of patients. Therefore, the time-varying brain activity characterized by dALFF may reveal the biological basis of neurological deficits in patients with ischemic stroke. The dynamics of brain activity in acute BGIS have not been quantified, and the mechanism of motor dysfunction reorganization is not yet clear.

This study applies dALFF to explore the characteristics of brain activity changes in acute patients with BGIS and elucidates its significance for motor function remodeling.

## Materials and methods

### Participants

From May 2019 to December 2022, 43 acute BGIS patients who had neurologic symptoms and have been judged by clinical neurologists were consecutively recruited from the Department of Neurology, the First Affiliated Hospital of Guangxi Medical University, with an ethics approval number 2021 (K Y-E-184) and application date of August 24, 2021. The inclusion criteria for all patients are as follows: (1) first onset acute BGIS diagnosed by a consensus of a clinical neurologist and a radiologist; (2) age between 30 and 75 years; (3) ischemic stroke accompanied with motor dysfunction onset within 10 days; (4) right-handedness before stroke. The exclusion criteria of this study included the following: (1) stroke lesion located at another brain region rather than the basal ganglia; (2) other neurological disorders that would affect the experiment, such as hemorrhage, multiple infarcts, or psychiatric diseases; (3) the modified Fazekas score > 1 (4) any contraindications for MRI, including pregnancy and metal implants; (5) excessive head motion during rs-fMRI scanning. This protocol was approved by the Ethics Committee of the First Affiliated Hospital of Guangxi Medical University. All participants signed a written informed consent before the study.

In this study, forty-seven healthy controls (HCs) matched for age and education with no physical diseases or history of psychiatric or neurologic disorders who were recruited from local community were also recruited through advertising at the same time. The Nine acute BGIS patients were excluded from the final analysis due to poor image quality (*n* = 1), excessive head motion more than 3.0 mm of any direction of x, y, z or greater than 3°of rotation at any direction during the scanning (*n* = 2), missing data (*n* = 1), and incomplete scanning of cerebellum (*n* = 5), leaving 34 acute BGIS patients in the final analysis. Three HCs were excluded from the final analysis due to poor image quality (*n* = 1), excessive head motion (*n* = 1), and incomplete scanning of cerebellum (*n* = 1), leaving 44 HCs in the final analysis.

### Clinical scale tests

Stroke severity and neurological deficits were assessed using the National Institutes of Health Stroke Scale (NIHSS) (Kwah and Diong, 2014), Barthel Index (BI) (Kasner, 2006) was used to evaluate the ability of daily living, and motor function was assessed by the Fugl-Meyer Assessment (FMA) (Gladstone et al., 2002). FMA contains two components that can evaluate the movements of the upper and lower limbs separately, and we use Upper Extremity_FMA (UE_FMA) and Lower Extremity_FMA (LE_FMA) to represent the assessment of the upper and lower limb respectively, and calculate the total score in Whole Extreme_ FMA (WE_FMA) indicates.

### MRI data acquisition

All MRI data were obtained by a 3.0 T MR scanner (SIEMENS MAGNETOM Prisma), which is equipped with a 64-channel head–neck combined coil. Foam pads were employed to minimize noise and head motion. The participants were required to shut their eyes quietly, keep their minds clear, and not think deeply about anything. The rs-fMRI was acquired using an echoplanar imaging (EPI) sequence with the following parameters: 186 whole-brain volumes for each participant; repetition time/echo time = 2000 ms/35 ms, flip angle =90°, field of view = 240 × 240 mm, voxel size = 2.6 × 2.6 × 3 mm, matrix = 64 × 64, gap = 0 mm, and slice number = 40. This session lasted for 6 min and 12 s. The anatomical 3D-MPRAG T1-weighted images (T1WI) were recorded by magnetization prepared rapid gradient echo: repetition time/echo time = 2,300 ms/2.98 ms, reverse time = 900 ms, FOV = 256 × 256 mm, voxel size = 1.0× 1.0 × 1.0 mm, matrix = 256 × 256, gap = 0 mm, and slice number = 176. This session lasted for 5 min and 21 s.

### RS-fMRI data preprocessing

All algorithms were implemented in Matlab R2017b working platform.^1^ Rs-fMRI data preprocessing and statistical analyses were carried out using the Resting-State fMRI Data Analysis Toolkit plus V1.24 (RESTplus V1.24, http://www.restfmri.net) and Statistical Parametric Mapping (SPM12, http://www.fil.ion.ucl.ac.uk/spm/software/spm12/); The preprocessing steps included the following: (1) removing the first 10 time points to make the longitudinal magnetization achieve steady-state and to let the participants get used to the scanning environment; (2) slice timing correction; (3) head motion correction. Head motion exceeded 3 mm or 3° rotation were excluded from subsequent analysis, the mean frame displacement (FD) was calculated for each subject; (4) the functional images were spatially normalized to the Montreal Neurological Institute (MNI) space *via* the deformation fields derived from new segmentation of structural images (resampling voxel size =3 mm × 3 mm × 3 mm); (5) spatial smoothing with a Gaussian kernel of 6 mm full-width at half-maximum (FWHM); (6) removing the linear trend of the time series; (7) regressing out nuisance variables, including the Friston−24 head motion parameters, polynomial trend, white matter signals, and cerebrospinal flow signals.

### dALFF calculation

The method of sliding time window is used to calculate dALFF. The window length is an open but important parameter in the static dynamics calculation based on sliding windows (Liao et al., 2019). Ideally, the length of a window should be small enough to detect potentially transient signals and large enough to analyse the lowest fluctuations of interest in the signals. Previous studies have shown that the window length of 50 TRs (100 s) is the best parameter to maintain the balance between capturing fast changing dynamic relationships (shorter windows) and achieving reliable brain activity estimation (longer windows) (Guo et al., 2019; Cui et al., 2020; Yao et al., 2020). On this basis, we chose 50 TRs (100 s) as the sliding window length. The time series was comprised of 176 TRs (352 s), and the window was shifted by one TRs (2 s). The full-length time series was then divided into 127 windows for each participant. For each sliding window, the ALFF map was obtained. The ALFF of each voxel was divided by the global mean ALFF value to normalize the global effects. To quantify temporal variations in dALFF, we computed the coefficient of variation [CV = standard deviation (SD) /mean] map over time for each subject (Li et al., 2019).

### Statistical analysis

All statistical analyses were performed with Statistical Product and Service Solutions 23.0 (SPSS 23.0, IBM, Armonk, NY, United States). The measurement data subject to normal distribution are expressed by mean ± standard deviation (x ± s), the measurement data subject to nonnormal distribution are expressed by median (upper and lower quartiles), and the count data are expressed by percentage (%). Demographic and clinical data were analyzed using the chi-squared test for sex. Two-sample t-tests were used for the other demographic characteristics. Differences were considered significant at *p*<0.05. Two-sample t-tests were employed for whole brain dALFF maps comparisons to analyze between patients with BGIS and HCs. Age, sex, and education level were entered as nuisance covariates. The resultant T-maps were conducted with Gaussian Random Field Theory (GRF) correction for multiple comparisons with voxel *p* < 0.05, cluster *p* < 0.05. For metric (dALFF) which shows acute BGIS related alterations, Spearman’s correlation analysis was used to assess their associations with clinical scales (NIHSS scores、FMA scores、and BI scores) of patients. The correlations were considered significant at a threshold of *p* < 0.05.

## Results

### Participants’ characteristics

Demographics and clinical data of the acute BGIS patients and HCs were calculated (Table 1). There were no significant differences in age (*t* = 0.450, *p* = 0.654), education level (*t* = −0.852, *p* = 0.397) between acute BGIS Patients and HCs, but in gender (*χ^2^* = 7.184, *p* = 0.007) between acute BGIS patients and HCs was statistically significant (Table 1).

**Table 1:** Demographic and clinical characteristics of the participants.

	BGIS (*n* = 34)	HCs (*n* = 44)	Statistical	*p* value
Age (years)	56.500 ± 10.999	55.340 ± 11.485	*t* = 0.450	0.654
Education (years)	11.500 ± 3.587	12.23 ± 3.06	*t* = −0.852	0.397
Gender (male/female)	25/9	19/25	*χ^2^* = 7.184	0.007^*^
NIHSS	3.25 (1.75,5.75)	–		
WE_FMA	82.5 (64,87.67)	–		
UE_FMA	54 (40,58.89)	–		
LE_FMA	27.1 (24.2,29.5)	–		
BI	78 (58.75,95)	–		

^﹡^*p*<0.05; BGIS, Basal ganglia ischemic stroke; HCs, healthy controls; NIHSS, National Institutes of Health Stroke Scale; FMA, Fugl-Meyer Assessment scale; UE_FMA, Fugl-Meyer Assessment scale of the upper limbs; LE_FMA, Fugl-Meyer Assessment scale of the lower limbs; WE_FMA, Fugl-Meyer Assessment scale of total score of upper and lower limbs; BI, Barthel Index.

### Temporal variability of ALFF differences between the acute BGIS patients and HCs

**Figure 1:**
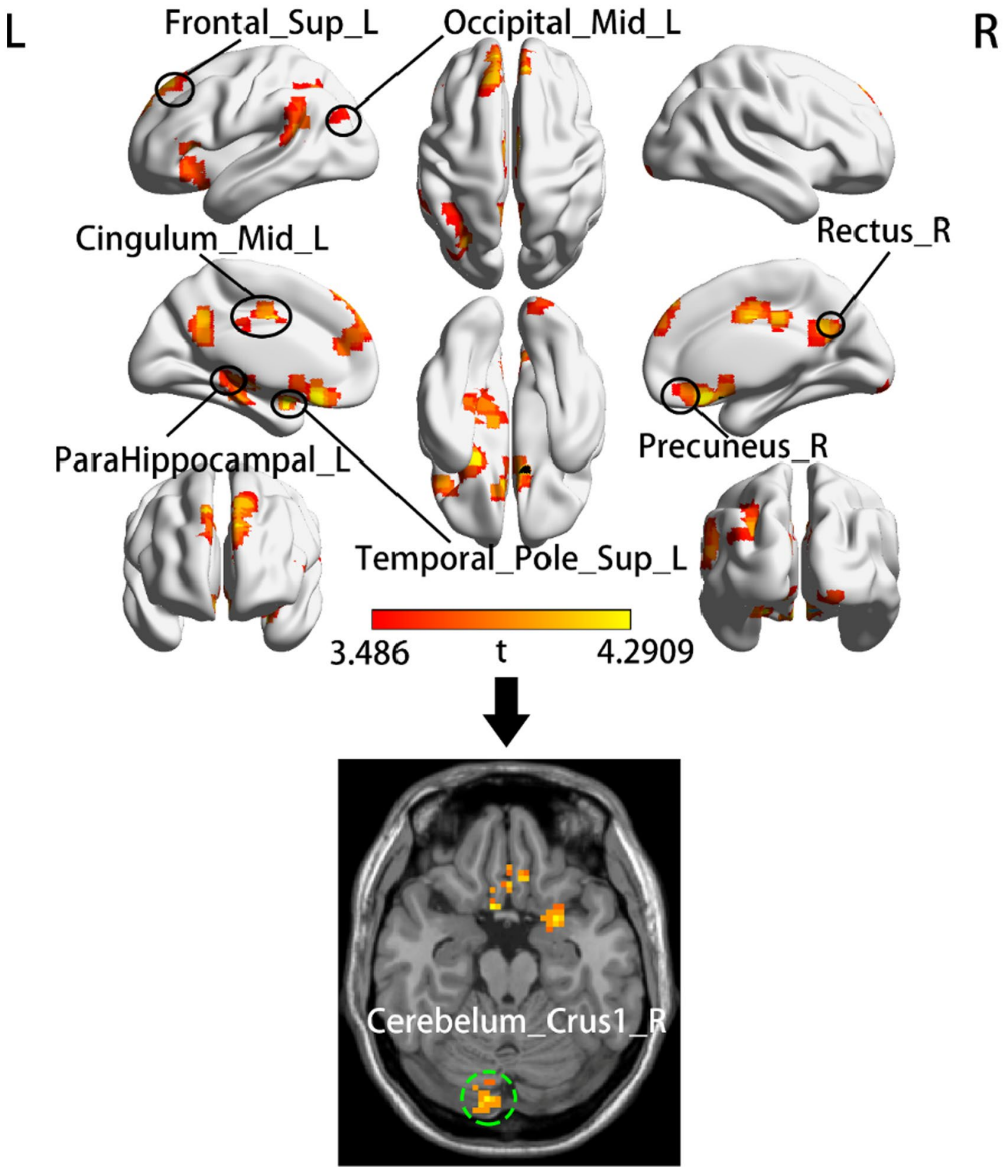
Group comparison of dALFF between the acute BGIS patients and HCs. The top half of the figure shows the clusters that located in the cerebral cortex and the bottom shows the cluster located in cerebellum. The red-yellow color represents positive *t*-statistics, which indicate significantly increased dALFF in the BGIS group compared to the group of HCs. *R*, right hemisphere; *L*, left hemisphere.

According to two-sample t tests, we found that increased dALFF variability in the left temporal pole (ITP), left parahippocampal gyrus (ParaHIP), left middle occipital gyrus (MOG), left dorsolateral superior frontal gyrus (SFGdl), right precuneus (PCu), right rectus, Mid-cingulate cortex (MCC) and right cerebellum crus1 was significantly different between the acute BGIS patients and HCs (GRF correction, voxel *p* < 0.05, cluster *p* < 0.05) (Table 2 and Figure 1).

**Table 2:** Brain regions showing dALFF differences between groups.

Regions (AAL)	BA	Cluster size	Peak *T* value	M N I (X, Y, Z)
Rectus_R	11	85	4.2909	9,12, −21
Temporal_Pole_Sup_L	38	86	4.2168	−24,6, −21
ParaHippocampal_L	34	61	3.6476	−36, −33, −3
Occipital_Mid_L	19	81	3.486	−33, −69, 33
Frontal_Sup_L	8/9	152	4.1757	−12,57, 39
Precuneus_R	7	80	3.7105	9, −54, 30
Cingulum_Mid_L	24	69	4.058	0, −9, 42
Cerebelum_Crus1_R	–	55	3.6150	−12, −90, −21

AAL, automated anatomical labeling; MNI, Montreal Neurological Institute; Rectus_R, right rectus; Temporal_Pole_Sup_L, left temporal pole; ParaHippocampal_L, left parahippocampal gyrus; Occipital_Mid_L, left middle occipital gyrus; Frontal_Sup_L, left dorsolateral superior frontal gyrus; Precuneus_R, right precuneus; Cingulum_Mid_L, left Mid-cingulate cortex; Cerebelum_Crus1_R, right cerebellum crus1. dALFF: dynamic Amplitude of low frequency fluctuation. The statistical threshold was set at voxel with *p* < 0.05 and cluster with *p* < 0.05 for multiple comparisons using Gaussian random field (GRF) theory corrected, cluster voxel ≥ 55.

### Relationship between local metrics and clinical scales

**Figure 2:**
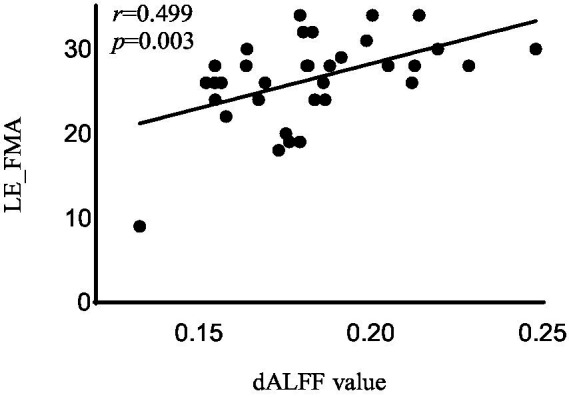
Correlation analysis between dALFF value and the LE_FMA scores in the right rectus in acute BGIS patients (*r* = 0.499, *p* = 0.003, *p* < 0.05). BGIS, basal ganglia ischemic stroke; dALFF, dynamic amplitude of low frequency fluctuation; LE_FMA, Fugl-Meyer Lower Extremity Assessment scale.

**Figure 3:**
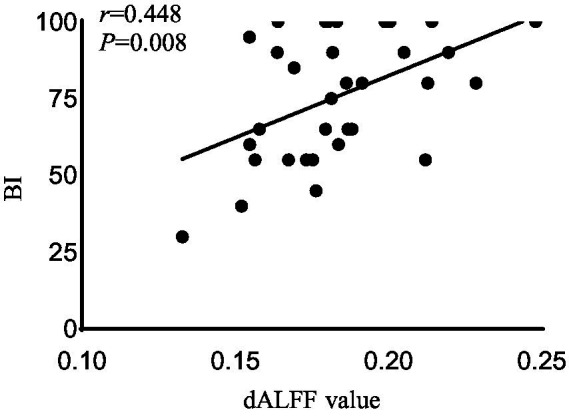
Correlation analysis between dALFF value and the BI scores in the right rectus in acute BGIS patients (*r* = 0.448, *p* = 0.008, *p* < 0.05). BGIS, basal ganglia ischemic stroke; dALFF, dynamic amplitude of low frequency fluctuation; BI, Barthel Index scale.

**Figure 4:**
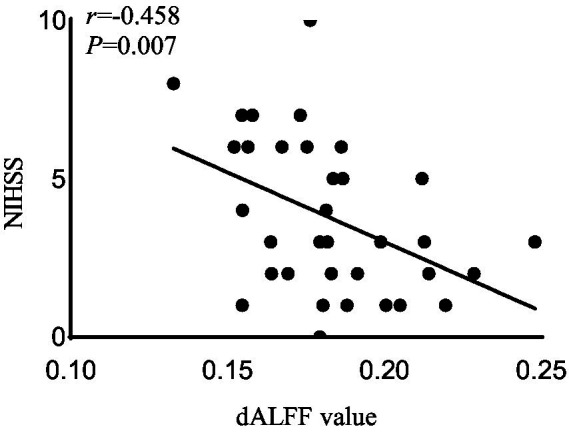
Correlation analysis between dALFF value and the NIHSS scores in the right rectus in acute BGIS patients (*r* = −0.458, *p* = 0.007, *p* < 0.05). BGIS, basal ganglia ischemic stroke; dALFF, dynamic amplitude of low frequency fluctuation; NIHSS, National Institutes of Health Stroke Scale.

In the acute BGIS patients, increased dALFF values of the right rectus were negatively correlated with NIHSS score (*r* = −0.458, *p* = 0.007) and positively correlated with LE_FMA score (*r* = 0.499, *p* = 0.003) and BI score (*r* = 0.448, *p* = 0.008) (Table 3 and Figures 2–4).

**Table 3:** The correlation results between brain regions and clinical measures.

		Correlation values			
Regions (AAL)	WE_FMA	UE_FMA	LE_FMA	BI	NIHSS
Rectus_R	*r* = 0.336	*r* = 0.299	*r* = 0.499	*r* = 0.448	*r* = −0.458
	*p* = 0.052	*p* = 0.086	*p* = 0.003^*^	*p* = 0.008^*^	*p* = 0.007^*^
Temporal_Pole_Sup_L	*r* = −0.083	*r* = −0.085	*r* = −0.069	*r* = −0.095	*r* = −0.014
	*p* = 0.641	*p* = 0.631	*p* = 0.697	*p* = 0.593	*p* = 0.559
ParaHippocampal_L	*r* = 0.190	*r* = 0.172	*r* = 0.182	*r* = 0.150	*r* = −0.134
	*p* = 0.282	*p* = 0.330	*p* = 0.302	*P* = 0.397	*p* = 0.452
Occipital_Mid_L	*r* = 0.144	*r* = −0.060	*r* = 0.037	*r* = 0.117	*r* = −0.028
	*p* = 0.415	*p* = 0.735	*p* = 0.834	*p* = 0.511	*p* = 0.873
Frontal_Sup_L	*r* = −0.043	*r* = 0.093	*r* = 0.245	*r* = 0.183	*r* = −0.149
	*P* = 0.415	*p* = 0.599	*p* = 0.163	*p* = 0.299	*p* = 0.399
Precuneus_R	*r* = −0.241	*r* = −0.253	*r* = −0.150	*r* = −0.145	*r* = 0.150
	*p* = 0.170	*p* = 0.150	*P* = 0.397	*p* = 0.413	*p* = 0.398
Cingulum_Mid_L	*r* = −0.310	*r* = −0.336	*r* = −0.235	*r* = −0.260	*r* = 0.191
	*p* = 0.074	*P* = 0.052	*p* = 0.180	*p* = 0.137	*p* = 0.279
Cerebelum_Crus1_R	*r* = 0.118	*r* = 0.093	*r* = 0.156	*r* = 0.104	*r* = 0.106
	*p* = 0.506	*p* = 0.601	*p* = 0.559	*P* = 0.559	*P* = 0.506

^*^p<0.05; AAL, automated anatomical labeling; NIHSS, National Institutes of Health Stroke Scale; FMA, Fugl-Meyer Assesment scale; UE_FMA, Fugl-Meyer Assesment scale of the upper limbs; LE_FMA, Fugl-Meyer Assesment scale of the lower limbs; WE_FMA, Fugl-Meyer Assesment scale of total score of upper and lower limbs; BI, Barthel Index. Rectus_R, right rectus; Temporal_Pole_Sup_L, left temporal pole; ParaHippocampal_L, left parahippocampal gyrus; Occipital_Mid_L, left middle occipital gyrus; Frontal_Sup_L, left dorsolateral superior frontal gyrus; Precuneus_R, right precuneus; Cingulum_Mid_L, left Mid-cingulate cortex; Cerebelum_Crus1_R, right cerebellum crus1.

### ROC analysis

**Figure 5:**
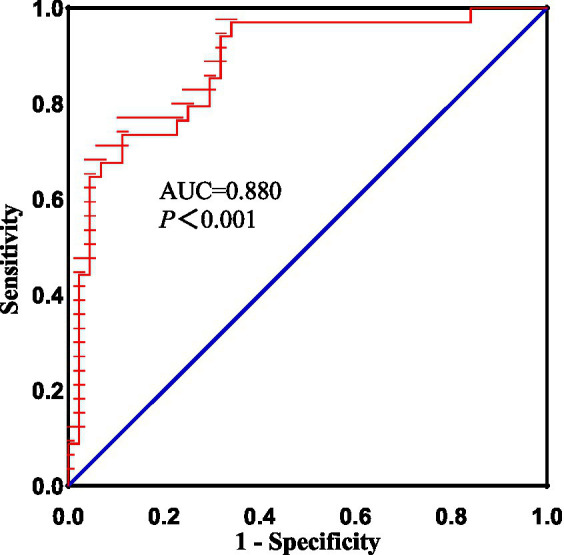
The diagnostic performance of altered dALFF in right rectus in distinguishing patients with acute BGIS from HCs. BGIS, basal ganglia ischemic stroke; dALFF, dynamic amplitude of low frequency fluctuation; AUC, area under curve.

As shown above, significant correlations were detected between the FMA scores 、BI scores、NIHISS scores, and dALFF variability in the right rectus, which proposed that the dALFF in the right rectus might be utilized to differentiate the patients with acute BGIS from HCs. To verify this possibility, ROC analysis was performed to investigate this possibility. The results demonstrated that the area under the curves (AUC) of the right rectus was 0.880, *p*<0.001 (Figure 5).

## Discussion

In this study, the method of sliding window was used to calculate dALFF for the first time to explore the time-domain dynamic characteristics of brain spontaneous activity and the relationship between the change of dALFF value and NIHSS, BI, and FMA scales in patients with acute BGIS with motor dysfunction. We found dynamic increase of activity in multiple regions in patients with acute BGIS, mainly in left TP, paraHIP, MOG, SFGdl, MCC, and right rectus, PCu, cerebellum crus1. In addition, we also found that the increased dALFF value in the right rectus was associated with BI, LE_ FMA was positively correlated and negatively correlated with NIHSS score. These findings suggest that the time-domain dynamic characteristics of spontaneous neuronal activity in specific brain regions may serve as potential neuroimaging markers for pathophysiological mechanisms in patients with acute BGIS.

Increased dALFF variability in the left MCC, MOG, and right cerebellum crus1 associated with motor information processing was observed in patients with acute BGIS. MCC is called the cingulate motor area. The middle cingulate cortex, thalamus, and basal ganglia are structurally and functionally related to each other and participate in somatic motor function (Amiez and Procyk, 2019). It has been found that electrical stimulation of the cortical area in the cingulate gyrus is sufficient to generate target oriented movement (Caruana et al., 2018). The cerebellum plays an important role in motor execution (Fujita, 2016; Huang et al., 2022). In our study, the cerebellum was activated in patients with acute BGIS with motor dysfunction, consistent with the reported results (Fu et al., 2016; Chen et al., 2018). The visual network is activated during the recovery of stroke movement, and limb movement is heavily dependent on visual guidance (Ward et al., 2003; Archer et al., 2016). The MOG may also be involved in the complex processing of movement (Li X. et al., 2020). Therefore, our results show that increased dALFF in the left MCC, MOG, and right cerebellum crus1 indicates increased variability and activity of local brain activity in these regions. This abnormal pattern indicates that patients with acute BGIS with motor impairment have the ability to reorganize motor function.

Previous studies have shown (Ward et al., 2003), that increasing attention to tasks may be a key strategy in the early stage of ischemic stroke when neurological deficits are greatest. SFGdl is located in the dorsolateral prefrontal cortex (DLPFC). It is connected to the middle frontal gyrus (MFG) and Inferior frontal gyrus (IFG) through arcuate fibers and participates in the execution of advanced complex cognitive tasks (Li et al., 2013).PCu is the key brain region of default mode network (DMN). DMN plays an important role in cognitive and emotional processing. Rs-FC studies have found that SFGdl is related to DMN, especially PCC/PCu function (Yu et al., 2011). Although DMN and central executive network (CEN) are considered to be two functionally antirelated networks, they are dynamically interactive and controlled and can effectively allocate attention (Leech et al., 2011). The SFGdl and PCu region also show increased dALFF variability. We speculate that the brain topology of patients with acute BGIS receives environmental stimulus feedback to cause local brain activity to conduct time self-organization (Gross and Blasius, 2008) and to compensate for motor function damage (Guo et al., 2020).

Patients with acute BGIS showed that the dALFF values in the limbic areas of the rectus, TP and paraHIP increased, which is a group of brain structures involved in emotion, motivation, learning, and memory, enabling the cerebral cortex to form a higher cognitive connection and procedural movement, achieving an ideal motor control effect (Chouinard and Paus, 2006). Some scholars believe that decreased activity is the expression of “low energy” and increased activity is the expression of “low efficiency,” that is, it requires extra effort to reach the normal level when performing tasks (Li et al., 2014). When a motor task requires more cognitive and sensory information processing, the higher brain center will gradually and more participate in it (Shimada et al., 2017). As verified by the above studies, the increased variability of spontaneous neuronal activity in the non-motor areas in this study may also be the compensation for motor injury in patients with acute BGIS. Thereafter, it is possible to utilize survival factors in the highly preserved nervous system to assist motor learning to facilitate the transition from attention dependent movement to more automated movement. In addition, our results also found that the dynamic increase of spontaneous neuronal activity in the early stroke stage was mainly concentrated in the second motor area (such as MCC and cerebellum). The results of this study are consistent with Li et al. (2016). Li et al.’s longitudinal study on BGIS found that during motor recovery, the signal activation area of cortical neurons gradually transferred from the second motor area to the main motor area.

In addition, the increase in dALFF values in the right rectus was associated with LE_ FMA and BI scores were positively correlated and negatively correlated with NIHSS scores, suggesting that the increased dALFF value in the right rectus may be an indicator related to the recovery of neurological function in patients with acute BGIS. The right rectus is related to human cognitive function. Previous studies have linked cognitive and motor functions with network connectivity, and found changes in network connectivity between the two under abnormal conditions (Wu et al., 2020; Temp et al., 2021). Our study shows that the dALFF value in this area is increased and is related to LE_ FMA scores were positively correlated. We speculate that higher cognitive and learning abilities may mediate better motor function recovery in patients with BGIS, which is consistent with the findings reported by Oveisgharan et al. (2018). The ROC analysis results demonstrated that the area under the curves (AUC) of the right rectus was 0.880, which suggested that dALFF value in the right rectus might have the potential to distinguish patients with acute BGIS from healthy subjects. The findings suggested that dynamic local brain activity may be a powerful neuroimaging indicator for pathophysiological mechanisms in acute BGIS and provide a new avenue to distinguish patients from the healthy population.

This study has several limitations: first, the sample size of this study is small, and a larger sample size is needed to confirm these results. Second, this study only included patients with acute BGIS. It can be imagined that a longer observation period may enrich and improve our understanding of the dynamic characteristics of spontaneous neuronal activity in the cerebral cortex during the recovery period of infarction.

## Conclusion

In conclusion, we found that early motor function remodeling in patients with acute BGIS occurred in multiple brain regions with dynamic intrinsic activity increased. The abnomal dALFF variability was correlated with symptomatology of motor dysfunction acute BGIS and contributed to distinguishing patients with acute BGIS from HCs. This study sheds new insight into the motor dysfunction underlying acute BGIS from the perspective of dynamic local brain activity and potentially informing the diagnosis of this disease.

## Data availability statement

The original contributions presented in the study are included in the article/supplementary material, further inquiries can be directed to the corresponding author.

## Ethics statement

The study was approved by the Ethics Committee of the First Affiliated Hospital, Guangxi Medical University, Nanning, China. The patients/participants provided their Functional magnetic resonance imaging of the brain written informed consent to participate in this study. Written informed consent was obtained from the individual(s) for the publication of any potentially identifiable images or data included in this article.

## Author contributions

All authors listed have made a substantial, direct, and intellectual contribution to the work and approved it for publication.

## Conflict of interest

The authors declare that the research was conducted in the absence of any commercial or financial relationships that could be construed as a potential conflict of interest.

## Publisher’s note

All claims expressed in this article are solely those of the authors and do not necessarily represent those of their affiliated organizations, or those of the publisher, the editors and the reviewers. Any product that may be evaluated in this article, or claim that may be made by its manufacturer, is not guaranteed or endorsed by the publisher.

## Footnotes

^1^ https://uk.mathworks.com/products/matlab
